# Promoter methylation of CDKN2A and lack of p16 expression characterize patients with hepatocellular carcinoma

**DOI:** 10.1186/1471-2407-10-317

**Published:** 2010-06-22

**Authors:** Antal Csepregi, Matthias PA Ebert, Christoph Röcken, Regine Schneider-Stock, Juliane Hoffmann, Hans-Ulrich Schulz, Albert Roessner, Peter Malfertheiner

**Affiliations:** 1 Department of Gastroenterology, Hepatology, and Infectious Diseases, Otto-von-Guericke University, 39120 Magdeburg, Germany; 2 Institute of Pathology, Otto-von-Guericke University, 39120 Magdeburg, Germany; 3 Department of Surgery, Otto-von-Guericke University, 39120 Magdeburg, Germany; 4 Department of Medicine, Hufeland Klinikum GmbH Bad Langensalza, 99947 Bad Langensalza, Germany; 5 Institute of Pathology, Christian-Albrecht University, 24105 Kiel, Germany; 6 Department of Medicine II, Klinikum rechts der Isar, Technical University, 81675 Munich, Germany; 7 Institute of Pathology, Friedrich-Alexander University of Erlangen-Nürnberg, 91054 Erlangen, Germany

## Abstract

**Background:**

The product of CDKN2A, p16 is an essential regulator of the cell cycle controlling the entry into the S-phase. Herein, we evaluated CDKN2A promoter methylation and p16 protein expression for the differentiation of hepatocellular carcinoma (HCC) from other liver tumors.

**Methods:**

Tumor and corresponding non-tumor liver tissue samples were obtained from 85 patients with liver tumors. CDKN2A promoter methylation was studied using MethyLight technique and methylation-specific PCR (MSP). In the MethyLight analysis, samples with ≥ 4% of PMR (percentage of methylated reference) were regarded as hypermethylated. p16 expression was evaluated by immunohistochemistry in tissue sections (n = 148) obtained from 81 patients using an immunoreactivity score (IRS) ranging from 0 (no expression) to 6 (strong expression).

**Results:**

Hypermethylation of the CDKN2A promoter was found in 23 HCCs (69.7%; mean PMR = 42.34 ± 27.8%), six (20.7%; mean PMR = 31.85 ± 18%) liver metastases and in the extralesional tissue of only one patient. Using MSP, 32% of the non-tumor (n = 85), 70% of the HCCs, 40% of the CCCs and 24% of the liver metastases were hypermethylated. Correspondingly, nuclear p16 expression was found immunohistochemically in five (10.9%, mean IRS = 0.5) HCCs, 23 (92%; mean IRS = 4.9) metastases and only occasionally in hepatocytes of non-lesional liver tissues (mean IRS = 1.2). The difference of CDKN2A-methylation and p16 protein expression between HCCs and liver metastases was statistically significant (p < 0.01, respectively).

**Conclusion:**

Promoter methylation of CDKN2A gene and lack of p16 expression characterize patients with HCC.

## Background

Hepatocellular carcinoma (HCC) shows a dramatic increase of its incidence worldwide over the last three decades [1] and is associated with a very poor prognosis [2,3]. Except for patients accessible for surgical therapy, the 5-year survival is less than 3% [2,3]. Chronic hepatitis B virus (HBV) with or without additional exposure to aflatoxin [4,5], chronic hepatitis C virus (HCV), and alcohol are the main risk factors of HCC [2,3,6]. Diagnosis is usually made at an advanced stage of the disease, and poorly differentiated tumors are sometimes a challenge for liver-pathologists.

Many types of cancers have an abnormal control of the transition from G_1 _to S phase of the cell cycle. CDKN2A is regarded as tumour suppressor gene because it is frequently silenced by deletion or inactivating mutation in human cancers [7-9]. The product of CDKN2A, p16 is a potent cyclin-dependent kinase inhibitor and a critical negative G1-specific regulator that halts cell-cycle progression at the G_1_-S-phase boundary. Loss of its function can lead to uncontrolled cell proliferation [7].

Hui et al. were the first to report that p16 is commonly deleted in HCC [10]. Initially, it was believed that inactivation of p16 in patients with primary liver cancer is related to homozygous deletions and mutations of CDKN2A gene [11-13]. Evidence is, however, increasing that gene silencing can also be achieved by methylation of CpG islands of the promoter region [14]. CDKN2A gene was reported to be extensively methylated in patients with HCC [15]. Indeed, methylation of CDKN2A was found not only in HCC, but also in patients with chronic liver disease [16,17] indicating that this finding may be non-specific for liver cancer. However, previous investigations used methylation-specific PCR (MSP) [18], a technique with limited sensitivity and specificity [19,20]. In our study, we investigated promoter methylation of CDKN2A using a novel sensitive method, MethyLight assay, in addition to the standard method of MSP, and p16 expression to identify and differentiate HCC from non-HCC liver tumors in a series of patients presenting with a liver mass.

## Methods

### Patients and tissue samples

Tumor and corresponding non-neoplastic liver tissue specimens were obtained from 85 patients who underwent surgical resection. Tissue samples were stored at - 80°C until analysis.

### Hepatocellular carcinoma (HCC)

Patients with HCC consisted of 28 men and 5 women ranging in age from 42 to 82 (median 67 ± 11.5) years. Mean tumor size was 75.1 ± 43.4 mm (range 15 to 230 mm). HCC samples were categorized according to differentiation into well (G1; seven cases), moderately (G2; nineteen cases), or poorly (G3; seven cases) differentiated types, which correspond to Edmondson's Grades I/II, III, and IV [21,22]. One patient (HCC patient No. 10) had a hepato-cholangiocellular carcinoma (Table 1). Regular intake of more than 60 g alcohol per day over a period of more than five years was documented in ten patients. Hereditary hemochromatosis was diagnosed in one, and chronic hepatitis C was found in ten patients. One patient (HCC patient No. 22) had a coinfection of HBV and HCV. Antibodies against HBV core protein were found in two patients (HCC patients No. 4 and No. 7) without evidence of viral replication. 13 tumors were classified as cryptogenic HCC (Table 1). Locally advanced disease was found in five cases at the time of liver resection. Alfa fetoprotein (AFP) serum level was available from 21 patients, ranging from 1.3 ng/ml to 95468 ng/ml (normal range < 7.0 ng/ml). In ten patients AFP level remained normal during follow-up. Advanced liver fibrosis or liver cirrhosis (fibrosis stages F3 to F6) according to the Ishak's scoring system [23] was confirmed in twenty-three patients (Table 1).

**Table 1:** Results and characteristics of patients with hepatocellular carcinoma (HCC)

Patient	Etiology	Non-tumor tissue					Tumor tissue			
		Fibrosis	Methylight		MSP	IRS	Methylight			
			**Result**	**PMR**			**Result**	**PMR**	**MSP**	**IRS**



HCC1	alcoholic	6	neg	0.08	neg	4	neg	0.14	neg	0
HCC2	cryptogenic	2	neg	0.04	neg	2	neg	3.73	pos	0
HCC3	HH	3	neg	0.01	pos	2	neg	0.04	pos	0
HCC4	alcoholic	3	neg	0.01	pos	0	neg	0.01	neg	0
HCC5	alcoholic	4	neg	1.39	neg	3	pos	100	neg	0
HCC6	cryptogenic	6	neg	0.19	pos	4	pos	14.64	pos	0
HCC7	cryptogenic	2	neg	0.01	neg	0	neg	0.41	pos	0
HCC8	alcoholic	2	neg	0.01	pos	0	pos	10.43	neg	0
HCC9	cryptogenic	5	neg	0.11	pos	4	pos	39.81	pos	0
HCC10	alcoholic*	4	neg	0.01	neg	2	pos	11.82	pos	4
HCC11	cryptogenic	2	neg	0.05	neg	2	neg	0.01	neg	3
HCC12	alcoholic	4	pos	22.44	neg	2	pos	26.86	pos	0
HCC13	alcoholic	4	neg	0.3	neg	0	pos	31.62	pos	0
HCC14	cryptogenic	1	neg	0.69	neg	3	pos	52.09	pos	0
HCC15	cryptogenic	2	neg	0.01	nd	0	pos	21.43	nd	0
HCC16	alcoholic	6	neg	0.71	pos	2	pos	100	pos	0
HCC17	cryptogenic	2	neg	0.12	nd	4	pos	22.11	nd	0
HCC18	cryptogenic	4	neg	0.15	neg	0	pos	41.06	pos	0
HCC19	alcoholic	6	neg	0.01	neg	0	pos	88.81	pos	0
HCC20	cryptogenic	2	neg	0.01	neg	2	neg	0.03	neg	0
HCC21	cryptogenic	3	neg	0.01	pos	0	pos	24.23	pos	2
HCC22	HBV/HCV	6	neg	1.46	neg	0	neg	1.92	neg	0
HCC23	HCV	1	neg	0.5	neg	0	pos	50.96	pos	0
HCC24	HCV	6	neg	0.86	neg	0	pos	7.91	pos	0
HCC25	HCV	6	neg	0.07	neg	0	neg	1.19	neg	0
HCC26	HCV	3	neg	0.19	pos	0	pos	74.13	pos	0
HCC27	HCV	6	neg	0.82	neg	0	neg	3.59	pos	0
HCC28	HCV	2	neg	2.31	neg	0	pos	11.14	pos	0
HCC29	HCV	6	neg	0.09	neg	0	pos	30.36	pos	0
HCC30	HCV	3	neg	0.19	neg	2	pos	19.78	pos	0
HCC31	HCV	5	neg	0.11	neg	0	pos	14.12	pos	4
HCC32	crpytogenic	2	neg	0.01	nd	nd	pos	88.22	nd	nd
HCC33	cryptogenic	6	neg	0.37	neg	nd	pos	100	neg	nd
HCC34	HCV	6	nd	nd	nd	0	nd	nd	nd	0
HCC35	HCV	6	nd	nd	nd	3	nd	nd	nd	0
HCC36	HCV/alcoholic	6	nd	nd	nd	0	nd	nd	nd	0
HCC37	HCV	5	nd	nd	nd	3	nd	nd	nd	0
HCC38	HCV	6	nd	nd	nd	3	nd	nd	nd	0
HCC39	HCV	5	nd	nd	nd	2	nd	nd	nd	0
HCC40	HCV	5	nd	nd	nd	6	nd	nd	nd	5
HCC41	HCV/alcoholic	3	nd	nd	nd	2	nd	nd	nd	0
HCC42	HCV/alcoholic	6	nd	nd	nd	1	nd	nd	nd	0
HCC43	HCV/alcoholic	na	nd	nd	nd	nd	nd	nd	nd	0
HCC44	HCV	na	nd	nd	nd	nd	nd	nd	nd	0
HCC45	HCV	na	nd	nd	nd	nd	nd	nd	nd	0
HCC46	HCV	na	nd	nd	nd	nd	nd	nd	nd	0
HCC47	HCV	na	nd	nd	nd	nd	nd	nd	nd	0
HCC48	HCV	na	nd	nd	nd	nd	nd	nd	nd	0

HCC, hepatocellular carcinoma; PMR, percentage of methylated reference (%); MSP, methylation specific PCR;

IRS = Immunoreactivity score, nd, not done; na, not available; AFP, alfa fetoprotein; * the tumor was considered as a mixed form of HCC and CCC; HBV, hepatitis B virus; HCV, hepatitis C virus;

### Bile duct cancer (CCC)

Tissue samples were obtained from 11 patients (4 men, 7 women) with adenocarcinoma of biliary tree and gall bladder with a mean age of 60.3 ± 8.1 years (range from 42 to 73 years) and graded as well (G1; one case), moderately (G2; eight cases), or poorly (G3; two cases) differentiated tumors. Two patients (CCC patients No. 9 and No. 10) developed advanced liver fibrosis (Table 2).

**Table 2:** Results and characteristics of patients with cholangiocellular carcinoma and benign liver tumors

Patient	Liver disease/	Non-tumor tissue					Tumor tissue			
	Primary cancer	Fibrosis	Methylight		MSP	IRS	Methylight		MSP	IRS
		**score**	**Result**	**PMR**			**Result**	**PMR**		

										
**Cholangiocellular carcinoma (CCC)**										
CCC1	bile duct	0	neg	0.01	pos	0	neg	0.01	neg	6
CCC2	bile duct	2	neg	0.01	neg	0	neg	0.04	neg	0
CCC3	bile duct	0	neg	0.01	neg	0	neg	0.01	pos	6
CCC4	bile duct	0	neg	0.01	neg	0	neg	0.8	pos	0
CCC5	gall bladder	0	neg	0.01	pos	0	pos	25.86	pos	2
CCC6	bile duct	0	neg	0.01	pos	2	neg	0.01	pos	2
CCC7	bile duct	2	neg	0.07	neg	0	neg	0.1	neg	5
CCC8	gall bladder	0	neg	0.01	neg	0	neg	3.28	neg	0
CCC9	bile duct	6	neg	0.01	neg	nd	neg	0.01	neg	nd
CCC10	bile duct	4	neg	0.49	neg	nd	neg	0.27	neg	nd
CCC11	gall bladder	1	neg	0.4	nd	nd	neg	0.01	nd	nd

										
**Benign liver tumors (B)**										
										
B1	FNH	0	neg	0.34	neg	0	neg	0.05	neg	2

B2	FNH	0	neg	0.05	neg	0	neg	0.25	neg	5

B3	hepatic adenoma	0	neg	0.01	nd	0	neg	0.01	nd	2

B4	FNH	0	neg	0.62	neg	2	neg	0.07	neg	0

B5	FNH	0	neg	0.1	nd	nd	neg	0.04	nd	nd

B6	FNH	0	neg	0.07	nd	nd	neg	1.36	nd	nd

B7	FNH	0	neg	0.03	nd	nd	neg	0.26	nd	nd

PMR, percentage of methylated reference (%); MSP, methylation specific PCR, IRS = Immunoreactivity score; nd, not done; CCC, cholangiocellular carcinoma; FNH, focal nodular hyperplasia;

### Benign liver tumors

Samples from seven patients (1 man, 6 women) with a benign liver tumor, i.e. focal nodular hyperplasia (n = 6) or liver adenoma (n = 1) were also studied (Table 2). Age of patients ranged from 22 to 47 (mean 40 ± 6) years. Advanced liver fibrosis was not detected in this patients' group.

### Liver metastases (M)

Tissue samples were also obtained from 34 patients (21 men, 13 women) with liver metastasis of colorectal (n = 29), gastric (n = 2), renal (n = 1), and breast (n = 2) cancers. Age of patients ranged from 32 to 82 (median 63.9 ± 8.2) years. Liver metastases were also categorized according to differentiation into well (G1; two case), moderately (G2; twenty-five cases), or poorly (G3; seven cases) differentiated carcinomas (Table 3). Advanced liver fibrosis (Ishak's stages F3 to F6) [23] was found only in one non-cancer liver tissue (M7) (Table 3).

**Table 3:** Results and characteristics of patients with liver metastasis

Patient	Primary cancer	Non-tumor tissue					Tumor tissue			
		Fibrosis	Methylight		MSP	IRS	Methylight		MSP	IRS
		score	Result	PMR			Result	PMR		
**Liver metastasis (M)**										
M1	breast	1	neg	0.05	nd	3	neg	0.02	nd	6
M2	breast	0	neg	0.04	nd	2	neg	0.01	nd	6
M3	colorectal	0	neg	0.01	nd	0	neg	0.01	nd	4
M4	colorectal	0	neg	0.01	nd	0	neg	0.01	nd	6
M5	colorectal	0	neg	0.01	nd	0	neg	0.01	nd	3
M6	colorectal	0	neg	0.01	nd	0	neg	0.01	nd	6
M7	colorectal	4	neg	0.01	nd	0	neg	0.01	nd	5
M8	colorectal	0	neg	0.01	nd	0	neg	0.01	nd	5
M9	colorectal	0	neg	0.01	nd	0	neg	0.01	nd	6
M10	colorectal	0	neg	0.01	nd	0	neg	0.01	nd	5
M11	colorectal	0	neg	0.01	nd	2	pos	39.55	nd	4
M12	colorectal	0	neg	0.01	nd	0	neg	0.01	nd	6
M13	colorectal	0	neg	0.01	neg	0	neg	0.01	neg	6
M14	colorectal	0	neg	0.02	pos	0	neg	0.01	pos	6
M15	colorectal	0	neg	0.01	neg	0	pos	52.18	neg	6
M16	colorectal	0	neg	0.09	neg	0	neg	0.74	neg	6
M17	colorectal	0	neg	0.39	neg	2	neg	0.04	neg	6
M18	colorectal	0	neg	0.73	neg	0	neg	0.12	neg	5
M19	colorectal	0	neg	0.15	neg	0	neg	0.01	neg	5
M20	colorectal	0	neg	0.17	pos	2	neg	0.01	neg	4
M21	colorectal	0	neg	0.8	neg	3	pos	57.82	pos	0
M22	colorectal	0	neg	0.54	pos	2	neg	0.35	neg	5
M23	colorectal	2	neg	0.55	pos	0	neg	0.51	neg	6
M24	colorectal	0	neg	0.38	neg	0	pos	9.88	pos	0
M25	colorectal	0	neg	0.08	pos	0	pos	6.48	neg	5
M26	gastric	0	neg	0.01	pos	nd	neg	0.01	neg	nd
M27	gastric	0	neg	0.39	neg	nd	neg	0.15	neg	nd
M28	colorectal	0	neg	0.09	neg	nd	pos	25.19	neg	nd
M29	colorectal	0	neg	0.85	pos	nd	neg	0.07	pos	nd
M30	colorectal	0	neg	0.86	neg	nd	neg	0.49	neg	nd
M31	colorectal	0	neg	0.55	pos	nd	neg	1.19	pos	nd
M32	renal	0	neg	0.01	nd	nd	neg	0.01	nd	nd
M33	colorectal	1	neg	0.08	nd	nd	neg	0.85	nd	nd
M34	colorectal	0	neg	0.03	nd	nd	neg	0.01	nd	nd

PMR, percentage of methylated reference (%); MSP, methylation specific PCR; nd, not done;

### Tissue samples for immunohistochemistry

Only tissue slides were available for immunohistochemical analysis from further 23 patients [HCC (n = 15) (Table 1) and chronic hepatitis C (n = 8)]. All the 15 HCC patients had a HCV-related chronic liver disease, and four consumed also regularly alcohol. Core antibodies against HBV were present in two patients. Liver cirrhosis was confirmed in six samples.

The study was approved by the Ethics Committee of the Otto-von-Guericke University of Magdeburg.

### DNA extraction and sodium bisulfit modification

Genomic DNA was extracted using proteinase K digestion and was modified by sodium bisulfit (CpGenome™ DNA Modification Kit, Q-Biogene Heidelberg, Germany) as reported previously [24,25].

### MethyLight assay

Genomic DNA was analyzed by MethyLight technique after bisulfite conversion as reported previously [19,20]. Briefly, three oligonucleotids were used in every reaction. Two locus-specific PCR primers flanked an oligonucleotide probe with a 5' fluorescent reported dye (6FAM) and a 3'quencher dye (BHQ-1). Primer and probe sequences for CDKN2A promoter (GeneBank accession number GI 21886808) were: forward primer (bp 20012-20033) 5'-tgg-agt-ttt-cgg-ttg-att-ggt-t-3'; reverse primer (bp 20081-20060): 5'-aac-aac-gcc-cgc-acc-tcc-c-3'; oligonucleotide probe sequence (5'-3') (bp 20060-20043): 6FAM-acc-cga-ccc-cga-acc-gcg-BHQ-1. PCR reaction resulted in a 70-bp product including 8 CpG islands localized on the first exon of the gene. The gene of interest was then amplified and normalized to a reference set (β-actin = ACTB) to normalize for input DNA. The specificity of reactions for methylated DNA was confirmed using CpGenome Universal Methylated DNA [(Chemicon International Inc., CA, USA (subsidiary or Serologicals) catalog #S7821)]. TaqMan PCR reactions were performed in parallel with primers specific for the bisulfite-converted methylated sequence for a particular locus and with ACTB reference primers. The ratio between values was calculated in these two TaqMan analyses, using this approach the degree of methylation at that locus was determined. The extent of methylation at a specific locus was determined by the following formula: [(gene/actb)^sample^: (gene/actb)^SssI-treated genomic DNA^]* 100. Samples with ≥ 4% of PMR (percentage of methylated reference) were regarded as hypermethylated.

### Methylation-specific PCR (MSP)

The methylation status of CDKN2A was also evaluated by MSP as described previously[18,26]. In brief, DNA was subjected to bisulfit modification and was amplified using two primers specific for either methylated or unmethylated sequences [18]. Primer sequences for unmethylated reactions were sense: 5'-tta-tta-gag-ggt-ggg-gtg-gat-tgt-3' and anti-sense: 5'-caa-ccc-caa-acc-aca-acc-ata-a-3', which amplified a 151 bp product. Primer sequences for methylated reactions were sense: 5'-tta-tta-gag-ggt-gcg-gat-cgc-3 and anti-sense: 5'-gac-ccc-gaa-ccg-cga-ccg-taa-3', which amplified a 150 bp product. CpGenome™ Universal Methylated DNA (Q-Biogene, Heidelberg, Germany) was used as positive control. DNA from normal lymphocytes served as negative control for methylated alleles. PCR was performed as described previously [26]. PCR products were run on polyacrylamide gels, followed by silver-staining.

### Immunohistochemistry

p16 expression was studied on formalin-fixed and paraffin embedded tissue samples. Briefly, 3-5 μm thick tissue sections were deparaffinized in xylol and rehydrated in a graded alcohol series. Endogenous biotin was blocked using the Avidin/Biotin Blocking kit (2 × 15 minutes; Vector Laboratories, Inc., Burlingame, CA). Immunostaining was performed with polyclonal anti-p16 antibody directed against the entire region of the human p16 protein (1:100; Quartett, Berlin, Germany). Incubation with the primary antibody was performed in a moist chamber at 37°C for 1 hour. Rabbit-anti-mouse IgG (30 minutes, room temperature; Vector Laboratories Inc.) served as a secondary antibody. The immunoreaction was visualized with an streptavidin-biotin complex, using the Vectastain ABC alkaline phosphatase kit (distributed by CAMON, Wiesbaden, Germany). FastRed (Zytomed, Berlin, Germany) served as chromogen. The specimens were counter-stained with hematoxylin. Omission of the primary antibody served as a negative control.

### Immunoreactivity Score (IRS)

For quantification of immunohistochemical results, a numerical scoring system was applied. Only nuclear staining was considered as positive. The observed expression of p16 in hepatocytes and tumor cells was assessed using two categories. Category A documented the number of immunoreactive cells as 0 (no reactive cells), 1 (< 10%), 2 (11 to 50%), and 3 (> 50%). Category B documented the intensity of immunostaining as 0 (no immunostaining), 1 (weak), 2 (moderate), and 3 (strong). Finally, values for category A and B were added to construct an "immunoreactivity score" (IRS) ranging from 0 to 6.

### Statistical analysis

PMR (percentage of methylated reference) values of Methylight assays were dichotomized for statistical purposes as reported previously [19,20]. PMR values of tumor and non-tumor samples were compared by Kolmogorov-Smirnov test. Differences in promoter methylation rate were analysed between cancer and non-tumor tissues using Fisher's exact test. All statistical tests were two-sided, with p < 0.05 considered statistically significant difference.

## Results

### MethyLight assay

**Figure 1:**
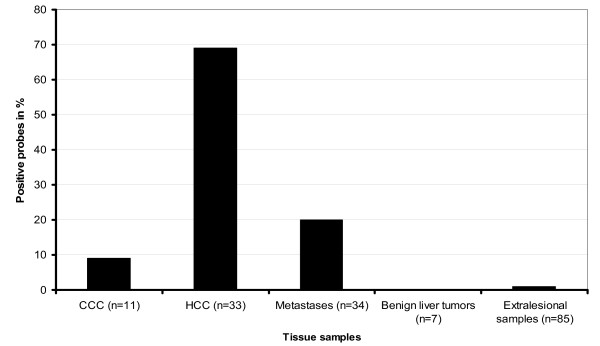
**Results of Methylight analysis**. Frequency of promoter methylation of CDKN2A gene is shown in patients with liver tumors detected by Methylight assay.

Using Methylight assay, methylation status of 8 CpG islands of the first exon of CDKN2A was evaluated in 33 HCCs and the corresponding non-tumor liver samples. The mean PMR of HCC samples was 31.41 ± 27.63% (range 0.01 to 100%) (Table 1). Twenty-three patients with HCC (69.7%) showed a PMR > 4% and were regarded as hypermethylated. The CDKN2A hypermethylation positive HCCs showed a mean PMR of 42.34 ± 27.8% (range 7.91 to 100%) (Table 1). A methylation of CpG islands was detected in 4 (57%) of 7 well-differentiated, 15 (79%) of 19 moderately differentiated, and 4 (57%) of 7 poorly differentiated HCCs. The frequency of promoter hypermethylation between patients with non-HCV induced HCC and those with HCV-induced HCC was not different (69% vs. 70%; ns). The mean PMR of the corresponding non-cancer liver tissues was 1.15 ± 1.64% (range 0.01 to 22.41). All but one (HCC patient No. 21) corresponding non-tumor samples obtained from patients with HCC were found to have an unmethylated CDKN2A promoter (Figure 1).

Next, we assessed the frequency of CDKN2A methylation in tumor and non-tumor tissue samples of 34 patients with liver metastases (Table 3) and 11 patients with adenocarcinomas of the biliary tract and gall bladder (CCC-group) as well as from 7 patients with benign liver tumors (Table 2).

None of tissue samples with benign liver pathology obtained from patients with non-HCC (altogether 59 samples including lesional samples of patients with benign liver tumors) showed CpG methylation. Liver metastases of 6 colorectal cancers (20.7%) had a mean PMR of 39.23% (range 25.18 to 52.18%). Only one tumor sample (9%) of the CCC-group had a PMR of 25.86% (Tables 2 and 3) (Figure 1). PMR in liver metastases was comparable to those results obtained from patients with HCC.

### MSP analysis

**Figure 2:**
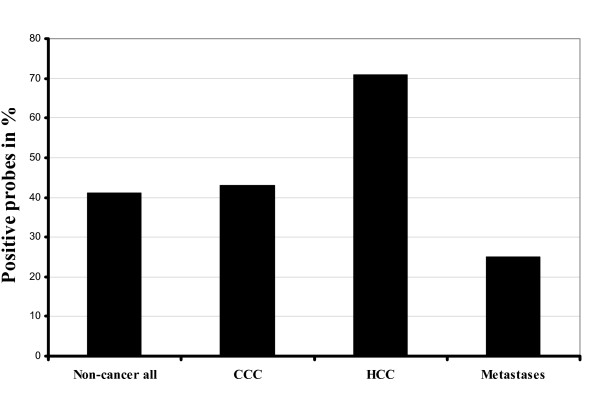
**Results of MSP analysis**. Frequency of promoter methylation of CDKN2A gene is shown in patients with liver tumors detected by MSP.

Tumor und non-tumor tissue samples from 30 patients with HCC were studied using MSP. 30% of the non-tumor and 70% of the tumor samples were found to be hypermethylated. There was no significant difference between the HCV-induced tissue probes (80%) and those from patients with HCC not related to HCV (65%) in terms of methylation of promoter region. 17 HCC samples were hypermethylated with both methods (MethyLight and MSP). None of the MSP-positive non-tumor samples showed hypermethylation analysed by MethyLight. Altogether, about half of cancer probes (21 HCCs, 1 CCC, 6 liver metastases) were found hypermethylated in MSP analysis. Among patients with MSP-positive metastatic colorectal cancer, only 2 (patients 21 and 24) showed a methylated promoter also in MethyLight analysis (Table 3). It was of interest that 32% of the non-neoplastic liver tissue samples proved methylated in MSP analysis (Figure 2).

### Immunohistochemical staining

**Figure 3:**
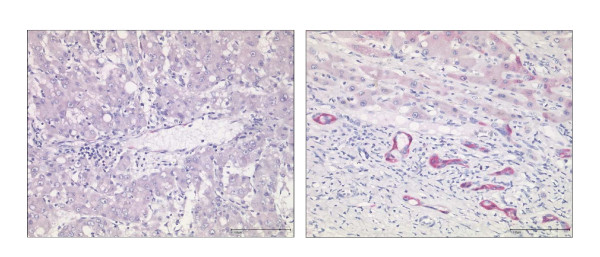
**Quantitative differences of p16 protein expression**. In liver tumors and non-neoplastic tissue samples the quantitative differences of p16 expression evaluated by immunohistochemical detection of p16 using an immunoreactivity score (IRS) are shown.

**Figure 4:**
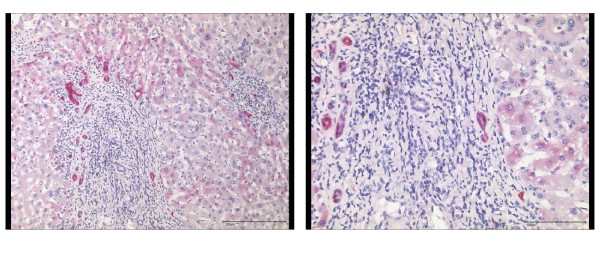
**Immunohistochemical expression of p16 in non-neoplastic liver tissue and hepatocellular carcinoma**. A moderately differentiated HCC is completely immunonegative for p16 (left). The non-neoplastic liver tissue shows immunostaining for p16 in scattered ductular reactions and mild cytoplasmic immunostaining in few periportal and periseptal hepatocytes (right). Anti-p16-antibody. Hematoxylin-counterstain.

**Figure 5:**
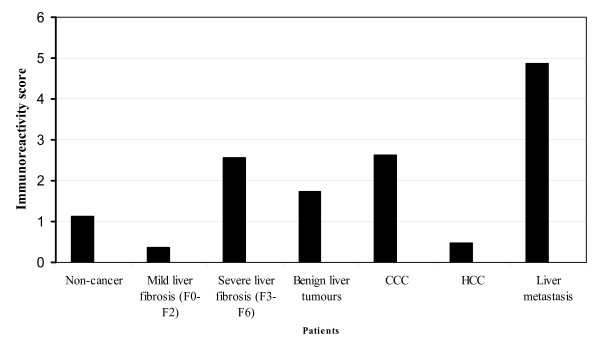
**Immunohistochemical expression of p16 in non-tumor liver sample**. The non-neoplastic liver tissue shows strong immunostaining for p16 in the ductular reactions and mild cytoplasmic immunostaining in few periportal hepatocytes. Anti-p16-antibody. Hematoxylin-counterstain.

**Figure 6:**
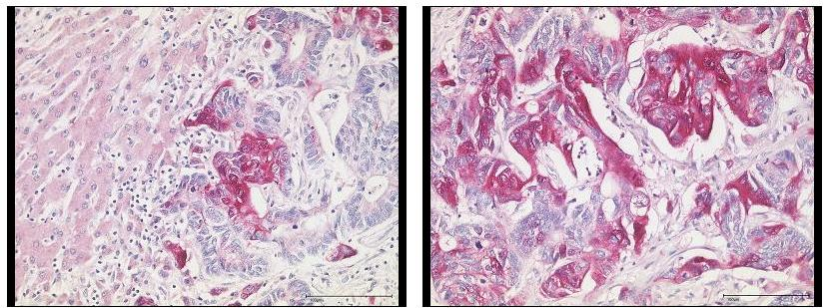
**Immunohistochemical detection of p16 in a liver metastasis**. Liver metastasis of a moderately differentiated colorectal cancer with strong cytoplasmic and also nuclear expression of p16 in tumor cells. Non-neoplastic liver tissue adjacent to colorectal cancer metastasis is completely devoid of p16 expression. Anti-p16-antibody. Hematoxylin-counterstain.

148 tissue sections were obtained from 81 patients [HCC (n = 36), CCC-group (n = 8), benign liver tumors (n = 4), liver metastasis (n = 25) and chronic hepatitis C (n = 8)] and were studied by immunohistochemistry for p16 protein expression. Non-cancer samples (n = 75) showed only a weak immunoreactivity for p16 (mean IRS: 1.24) in few hepatocytes (Figures 3 and 4). While samples (n = 45) without significant liver fibrosis (F0 to F2) expressed no p16 (mean IRS: 0.36), immunostaining was significantly enhanced in samples (n = 30) with advanced liver fibrosis or cirrhosis (F3 to F6) (mean IRS: 2.6) (p < 0.01) (Figure 5). Here, p16 was mainly expressed by hepatocytes lining the portal tracts and fibrous septa as well as by cells of ductular proliferations. Nuclear p16 expression was absent in the vast majority of HCC samples (Figure 3). Only 5 samples (11%, mean IRS: 0.47) obtained from moderately differentiated HCCs expressed p16 in the nucleus. In contrast, metastatic cancer probes (23/25) showed a strong p16 expression (mean IRS: 4.9) (p < 0.01) (Figure 6). A complete loss of nuclear p16 expression was seen only in two remaining samples from patients with colorectal cancer (patients 21 and 24) (Table 3). Tissue samples obtained from patients of the CCC-group (IRS: 2.6) or with benign liver tumors (IRS: 1.75) showed only a moderate p16 immunostaining.

The difference of CDKN2A-methylation and p16 protein expression between HCCs and liver metastases was statistically significant (p < 0.01, respectively). Using the Spearman non-parametric test, no correlation between nuclear p16 expression detected by immunohistochemical staining and CDKN2A promoter methylation using Methylight assay could be shown.

## Discussion

CDKN2A encodes a cyclin-dependent kinase inhibitor, a negative G_1_-specific regulator of cell cycle and was reported to be inactivated through extensive CpG methylation in hepatitis virus-induced HCC [15]. However, CpG islands of CDKN2A were shown to be also methylated using MSP [18], a technique having limited sensitivity and specificity [19,20], in patients with chronic liver diseases without HCC [16,17]. In order to further clarify this issue, in the first step of our analysis, we focused on epigenetic changes of CDKN2A evaluating the methylation status of CpG islands on the first exon. MethyLight provides a sensitive tool for detecting CpG methylation [19,20] and also allows to quantitatively assess the grade of methylation. Our findings indicate that methylation of CpG islands at the 5'end of the first exon of CDKN2A detected by MethyLight, but not by MSP, may serve as a specific marker of HCC since it was frequently found in HCCs and detected only in one non-cancer sample. Epigenetic changes of CpG islands are probably related not only to chronic viral hepatitis as suggested in several previous reports [16,17,27]. In our study patients with non-HCV induced HCC showed the same frequency of promoter hypermethylation as HCV induced HCCs. Hypermethylation of CDKN2A gene was not found in any of the extralesional liver tissue samples obtained from patients with chronic hepatitis C. 20% of patients with liver metastases of colorectal cancers had methylated CpG islands and that frequency is somewhat lower than reported in the literature [25,28,29].

In previous studies it was shown that p16 was expressed at very low levels in mammalian tissues suggesting that this protein may have no impact on the normal development of cells [30,31]. In our study, non-cancer liver samples without advanced fibrosis failed to express p16 in the nucleus suggesting that p16 is expressed only at low levels in non-neoplastic tissue. In contrast, in patients with severe liver fibrosis or cirrhosis which are considered as a preneoplastic condition for HCC, an increased nuclear p16 expression was observed. Nuclear p16 expression was not detected in the majority of HCCs indicating that loss of p16 may be a common event during hepatocarcinogensis [15]. The absence of p16 expression resulting from either methylation or mutation of CDKN2A gene may probably reflect increased selection pressure at this neoplastic stage for loss of p16 expression. In our study, we found no correlation between methylation of the first exon of CDKN2A gene and nuclear expression of p16.

Data on p16 protein expression in liver metastases are scarce. Dai et al reported [32] that 89% of colon adenomas, 65% of colon carcinomas and 100% of liver metastasis of colon cancer expressed p16. In line with these results, liver metastases from colon cancers showed also in our study a strong p16 expression. Overexpression of p16 in metastatic tissue samples appeared to be paradoxical and was seen in several primary gastrointestinal tumors [32-34]. The precise reason why negative regulators of the cell cycle often display increased expression is unknown at present and remains to be elucidated.

## Conclusions

In summary, using MethyLight assay for epigenetic studies of CDKN2A in patients with liver tumors we showed that CDKN2A methylation is a putative marker of HCC. MSP compared to MethyLight seems to be unspecific in patients with liver tumors.

Our data also indicate that the combined analysis of CDKN2A methylation and immunohistochemical staining for p16 expression might be an approach to distinguish HCC from non-HCC liver tumors and liver metastases. Moreover, it is of particular interest that p16 expression seems to correlate with the grade of liver fibrosis. This finding deserves further studies.

## Competing interests

The authors declare that they have no competing interests.

## Authors' contributions

AC carried out methylation studies, analysis of data, drafted the manuscript and performed the statistical analysis. MPAE participated in its design and coordination und drafted the manuscript. CR carried out immunohistochemical studies und participated in its design and analysis. RSS participated in its design and coordination. JH carried out MethyLight analysis. HUS participated in the analysis of the study and drafted the manuscript. AR edited the manuscript. PM drafted the manuscript and participated in its coordination. All authors read and approved the final manuscript.

## Pre-publication history

The pre-publication history for this paper can be accessed here:

http://www.biomedcentral.com/1471-2407/10/317/prepub
